# *Rickettsia felis* in *Xenopsylla cheopis*, Java, Indonesia

**DOI:** 10.3201/eid1208.060327

**Published:** 2006-08

**Authors:** Ju Jiang, Djoko W. Soeatmadji, Katherine M. Henry, Sutanti Ratiwayanto, Michael J. Bangs, Allen L. Richards

**Affiliations:** * US Naval Medical Research Center, Silver Spring, Maryland, USA;; † Brawijaya University, Malang, Indonesia;; ‡ US Naval Medical Research Unit #2, Jakarta, Indonesia

**Keywords:** Rickettsia felis, Xenopsylla cheopis, Indonesia, flea-borne spotted fever, murine typhus, Rickettsia typhi, rodents

## Abstract

Rickettsia typhi and R. felis, etiologic agents of murine typhus and fleaborne spotted fever, respectively, were detected in Oriental rat fleas (Xenopsylla cheopis) collected from rodents and shrews in Java, Indonesia. We describe the first evidence of R. felis in Indonesia and naturally occurring R. felis in Oriental rat fleas.

Murine typhus (endemic typhus, fleaborne typhus), caused by *Rickettsia typhi*, is transmitted to humans by infected fleas and is relatively common wherever susceptible rodent hosts reside (*1*). Fleaborne spotted fever (cat flea typhus), caused by *Rickettsia felis*, is another zoonotic disease carried by fleas and appears to have an equally wide, cosmopolitan distribution; human infections with *R. felis* have a clinical syndrome similar to that of murine typhus (*1**–**4*). The cat flea, *Ctenocephalides felis*, has been identified as the primary arthropod vector of *R. felis* in North and South America (United States, Mexico, Peru, Brazil), Europe (Spain, France, United Kingdom, Cyprus), Africa (Gabon, Ethiopia), Asia (Thailand, Afghanistan, Israel), Australia, and New Zealand (*1**,**5**–**10*). We describe the first evidence of *R. felis* in Indonesia and apparent natural infections of *R. felis* in the Oriental rat flea, *Xenopsylla cheopis*, implicating this flea species for the first time as a potential vector for fleaborne spotted fever.

## The Study

Samples of *X. cheopis* were collected from 39 live-captured, peridomestic rodents and shrews from 3 localities in Malang, East Java, Indonesia, during an epidemiologic study conducted in 1994 (*11*). In this study the fleas were reidentified by using morphologic criteria, stored in fresh 70% ethanol, and subsequently evaluated for the presence of rickettsial DNA. DNA sample preparations were derived from triturates of 103 individual fleas in 100 μL PrepManUltra sample preparation reagent (Applied Biosystems, Foster City, CA, USA). DNA preparations of 1 to 5 fleas collected from the same rodent were pooled for testing. Reaction mixtures for the quantitative real-time PCR (qPCR) assays had a total volume of 25 μL and contained 3 μL DNA template. The master mixes were prepared for the 17-kDa *Rickettsia*–, *R. typhi*– and *R. felis*–specific qPCR assays in a separate, clean (DNA-free) room as previously described (*6**,**12*). The primer and probe sequences for the 17-kDa *Rickettsia*-specific and *R. felis*–specific assays have been reported (*6**,**11*). The *R. typhi* forward (Rt557F: 5´-TGG TAT TAC TGC TCA ACA AGC T-3´) and reverse (Rt678R: 5´-CAG TAA AGT CTA TTG ATC CTA CAC C-3´) primers and probe (Rt640BP: 5´-TET-CGC GAT CGT TAA TAG CAG CAC CAG CAT TAT CGC G-DABCYL-3´) sequences are listed here. Included in each run were 3 negative controls (GIBCO Ultrapure DNA-free distilled water, Invitrogen Corporation, Grand Island, NY, USA), 1 produced in the clean room and 2 in a biosafety cabinet in another laboratory where DNA templates were added. A TOPO TA plasmid (Invitrogen Corporation) that contained the target sequence at 10^3^ copies for each assay was used as a positive control. qPCR reactions were incubated in the SmartCycler (Cepheid, Sunnyvale, CA, USA) at 94°C for 2 min, followed by 50 cycles of a 2-step amplification protocol of 94°C for 5 s and 60°C for 30 s. Fluorescence was monitored during the annealing step of each cycle, and data were analyzed with SmartCycler software version 2.0c (Cepheid).

Rickettsial DNA was detected by 17-kDa qPCR in 7 of 39 pools containing 1–5 *X. cheopis* fleas. To determine whether *R. typhi* or *R. felis* infected these fleas, PCR assays specific for *R. typhi* and *R. felis ompB* partial sequence targets were performed (Table). Results of these assays showed that 5 of the 7 *Rickettsia*-positive *X. cheopis* fleas were infected with *R. typhi*, and 2 were positive for *R. felis*. The remainder of the 32 pools and all nontemplate controls were negative for *R. typhi* and *R. felis*. Additionally, 15 *Rickettsia*-free *C. felis* fleas (Heska Corporation, Loveland, CO, USA), evaluated at the same time and under the same conditions as the Malang fleas, were negative for *R. typhi* and *R. felis*.

**Table:** Detection of Rickettsia typhi and Rickettsia felis in flea pools by quantitative, real-time PCR, East Java, Indonesia

Location and distribution of rodents and shrews		No. flea pools (total no. fleas)*	No. positive pools (%)		
			17 kDa	R. typhi	R. felis
Rural (Mulyorejo)					
	Rattus argentiventer (7.1%)	1 (2)	0	0	0
	Rattus rattus (14.3%)	2 (3)	0	0	0
	Rattus tiomanicus (57.1%)	1 (1)	0	0	0
	Mus musculus (10.7%)	1 (2)	0	0	0
	Chiropodomys gliroides (3.6%)	0			
	Suncus murinus (7.1%)	0			
	Total rural	5 (8)	0	0	0
Suburban (Bandungrejosari)					
	Rattus exulans (3.2%)	1 (3)	0	0	0
	R. rattus (74.2%)	13 (32)	1	0	1
	R. tiomanicus (19.4%)	4 (9)	0	0	0
	C. gliroides (3.2%)	1 (4)	0	0	0
	Total suburban	19 (48)	1 (5.3)	0	1 (5.3)
Urban (Klojen)					
	R. exulans (5.3%)	1 (4)	1	1	0
	R. rattus (68.4%)	9 (28)	4	3	1
	R. tiomanicus (10.5%)	2 (7)	0	0	0
	Rattus sabanus (10.5%)	2 (5)	0	0	0
	M. musculus (5.3%)	1 (3)	1	1	0
	Total urban	15 (47)	6 (40)	5 (33.3)	1 (6.7)
Total		39 (103)	7 (18)	5 (12.8)	2 (5.1)

*Each pool of 1 to 5 fleas (Xenopsylla cheopis) came from a single animal.

## Conclusions

To determine the identity of rickettsial agents infecting *X. cheopis* fleas collected from rodents and shrews in Malang, we assessed pools of 1 to 5 fleas from each animal. Our results confirm *R. typhi* in a known flea vector of murine typhus in a highly disease-endemic region of East Java, Indonesia (*11**,**13*). *R. felis* has been shown to infect fleas of peridomestic rodents (*7**,**8*) and fleas other than *C. felis* (*1**,**5**,**14*). However our report is the first of *R. felis* naturally infecting *X. cheopis* fleas, a vector of plague and murine typhus. Both *R. felis*–containing flea pools were derived from *Rattus rattus*, 1 from the suburban and 1 from the urban neighborhoods of Malang. *R. rattus* was the predominant species captured in urban and suburban environments (72%) and appears to be the primary host for *R. felis*– and *R. typhi*– infected *X. cheopis*. In the rural setting, where *R. rattus* was represented with far less frequency (14.3%), neither rickettsial agent was detected in collected fleas. These findings merit further epidemiologic investigation to better understand the relationship between *R. felis*, *R. typhi*, and *X. cheopis* and the transmission dynamics between flea and rodent.

Additionally, this report provides the first evidence of *R. felis* in the Indonesian archipelago. Investigations of rickettsial agents in Indonesia have been relatively few; to date, human infections with *R. felis* have not been reported from Indonesia. The lack of reports may be because a murine typhus–like disease associated with *R. felis* infection would not allow healthcare providers to clinically discriminate fleaborne spotted fever from murine typhus or other rickettsioses. In Indonesia, rickettsioses and typhoid fever are collectively referred to as *tifus*. Rickettsial tifus can be discerned by serologic tests or by observing when rickettsial tifus cases rapidly respond to treatment with a tetracycline or chloramphenicol. Furthermore, the inability to diagnose fleaborne spotted fever by laboratory means has been attributed to the cross-reactivity of antibodies to *R. felis* antigens with other rickettsial antigens (*1*). Consequently, serologic assays have been unable to differentiate fleaborne spotted fever from other rickettsioses. Thus, the high prevalence of murine typhus reported in Indonesia likely also includes fleaborne spotted fever. In addition, previously demonstrated serologic evidence of spotted fever group rickettsiae infection among residents of Gag Island, in eastern Indonesia (*15*), could have been due to *R. felis*. On the basis of data presented here and of recent reports of *R. felis* in other countries in Asia (*2**–**5**,**8**,**9*), healthcare providers in Indonesia should be alerted to the possibility of fleaborne spotted fever among their patients.

## References

[1] Azad AF, Radulovic S. Higgins, Noden BH, Troyer JM. Flea-borne rickettsioses: ecological considerations. Emerg Infect Dis. 1997;3:319–27. 10.3201/eid0303.970308 9284376 PMC2627639

[2] Parola P, Miller RS, McDaniel P, Telford SRIII, Rolain J-M, Wongsrichanalai C, Emerging rickettsioses of the Thai-Myanmar border. Emerg Infect Dis. 2003;9:592–5. 12737744 10.3201/eid0905.020511 PMC2972759

[3] Choi Y-J, Jang W-J, Kim J-H, Ryu J-S, Lee S-H, Park K-H, Spotted fever group and typhus group rickettsioses in humans, South Korea. Emerg Infect Dis. 2005;11:237–44. 15752441 10.3201/eid1102.040603 PMC3320442

[4] Phongmany S, Rolain J-M, Phetsouvanh R, Blacksell SD, Soukkhaseum V, Rasachack B, Rickettsial infections and fever, Vientiane, Laos. Emerg Infect Dis. 2006;12:256–62. 16494751 10.3201/eid1202.050900 PMC3373100

[5] Parola P, Sanogo OY, Lerdthusnee K, Zeaiter Z, Chauvancy G, Gonzalez JP, Identification of *Rickettsia* spp. and *Bartonella* spp. in fleas from the Thai-Myanmar border. Ann N Y Acad Sci. 2003;990:173–81. 10.1111/j.1749-6632.2003.tb07359.x 12860622

[6] Blair PJ, Jiang J, Schoeler GB, Moron C, Anaya E, Céspedes M, Characterization of spotted fever group rickettsiae in flea and tick specimens from northern Peru. J Clin Microbiol. 2004;42:4961–7. 10.1128/JCM.42.11.4961-4967.2004 15528680 PMC525230

[7] Psaroulaki A, Antoniou M, Papaeustathiou A, Toumazos P, Loukaides F, Tselentis Y. First detection of *Rickettsia felis* in *Ctenocephalides felis* fleas parasitizing rats in Cyprus. Am J Trop Med Hyg. 2006;74:120–2. 16407355

[8] Marié J-L, Fournier P-E, Rolain J-M, Briolant S, Davoust B, Raoult D. Molecular detection of *Bartonella quintana, B. elizabethae, B. koehlerae, B. doshiae, B. taylorii*, and *Rickettsia felis* in rodent fleas collected in Kabul, Afghanistan. Am J Trop Med Hyg. 2006;74:436–9. 16525103

[9] Bauer O, Baneth G, Eshkol T, Shaw SE, Harrus S. Polygenic detection of *Rickettsia felis* in cat fleas (*Ctenocephalides felis*) from Israel. Am J Trop Med Hyg. 2006;74:444–8. 16525105

[10] Schloderer D, Owen H, Clark P, Stenos J. Fenwick. *Rickettsia felis* in fleas, Western Australia. Emerg Infect Dis. 2006;12:841–3. 16704850 10.3201/eid1205.051458 PMC3374419

[11] Richards AL, Soeatmandji DW, Widodo MA, Sardjono TW, Yanuwiadi B, Hernowati TE, Seroepidemiological evidence for murine and scrub typhus in Malang, Indonesia. Am J Trop Med Hyg. 1997;57:91–5. 9242326 10.4269/ajtmh.1997.57.91

[12] Jiang J, Chan TC, Temenak JJ, Dasch GA, Ching WM, Richards AL. Development of a quantitative real-time polymerase chain reaction assay specific for *Orientia tsutsugamushi.* Am J Trop Med Hyg. 2004;70:351–6. 15100446

[13] Corwin AL, Soepranto W, Widodo PS, Rahardjo E, Kelly DJ, Dasch GA, Short report: surveillance of rickettsial infections in Indonesian military personnel during peace keeping operations in Cambodia. Am J Trop Med Hyg. 1997;57:569–70. 9392597 10.4269/ajtmh.1997.57.569

[14] Stevenson HL, Labruna MB, Montenieri JA, Kosoy MY, Gage KL, Walker DH. Detection of *Rickettsia felis* in a New World flea species, *Anomiopsyllus nudata* (Siphonaptera: Ctenophthalmidae). J Med Entomol. 2005;42:163–7. 10.1603/0022-2585(2005)042[0163:DORFIA]2.0.CO;2 15799525

[15] Richards AL, Ratiwayanto S, Rahardjo E, Kelly DJ, Dasch GA, Fryauff DJ, Evidence of infection with ehrlichiae and spotted fever group rickettsiae among residents of Gag Island, Indonesia. Am J Trop Med Hyg. 2003;68:480–4. 12875301

